# MergedTrie: Efficient textual indexing

**DOI:** 10.1371/journal.pone.0215288

**Published:** 2019-04-23

**Authors:** Antonio Ferrández, Jesús Peral

**Affiliations:** 1 GPLSI Research Group, Department of Software and Computing Systems, University of Alicante, Alicante, Spain; 2 Lucentia Research Group, Department of Software and Computing Systems, University of Alicante, Alicante, Spain; Indian Institute of Technology Madras, INDIA

## Abstract

The accessing and processing of textual information (i.e. the storing and querying of a set of strings) is especially important for many current applications (e.g. information retrieval and social networks), especially when working in the fields of Big Data or IoT, which require the handling of very large string dictionaries. Typical data structures for textual indexing are Hash Tables and some variants of Tries such as the Double Trie (DT). In this paper, we propose an extension of the DT that we have called *MergedTrie*. It improves the DT compression by merging both Tries into a single and by segmenting the indexed term into two fixed length parts in order to balance the new Trie. Thus, a higher overlapping of both prefixes and suffixes is obtained. Moreover, we propose a new implementation of Tries that achieves better compression rates than the Double-Array representation usually chosen for implementing Tries. Our proposal also overcomes the limitation of static implementations that does not allow insertions and updates in their compact representations. Finally, our MergedTrie implementation experimentally improves the efficiency of the Hash Tables, the DTs, the Double-Array, the Crit-bit, the Directed Acyclic Word Graphs (DAWG), and the Acyclic Deterministic Finite Automata (ADFA) data structures, requiring less space than the original text to be indexed.

## 1. Introduction and motivation

Enormous amounts of data already exist and this is still rapidly growing due to diverse data sources such as sensors and social networks. There has been increasing interest in incorporating these huge amounts of external data, normally referred to as Big Data, into Smart Cities, Business Intelligence or IoT applications [1]. A large percentage of this information is unstructured and textual, such as that generated by social networks, whose information is especially critical for industrial companies nowadays, and which is extracted though Sentiment Analysis applications [2,3]. This growth boosts the development and researching of new Natural Language Processing (NLP) techniques in order to process efficiently textual information, which is especially crucial in Big Data applications (e.g. the biomedical text and data mining framework proposed in [4]) or auxiliary tasks such as Name Ambiguity Resolution [5], where the data sets (especially string dictionaries) are so large and complex that they become difficult to process using traditional data processing applications [6].

Modern NLP algorithms are often based on machine learning techniques that learn through the analysis of large corpora of real-world examples [7]. For example, statistical machine translation systems require parallel corpora formed by millions of sentence pairs, which are often too large to fit entirely into the main memory [8].

Information Retrieval (IR), currently popularized by Web search engines such as Google, is one of the applications that requires intensive text processing [9,10]. An IR system takes a user’s query as input and returns a set of documents sorted by their relevance to the query. IR systems are usually based on the segmentation of documents and queries into index terms or words, and that is why this kind of application is categorized as *term-level* or *word-level indexes*. The process of harvesting and indexing information to offer advanced search and discovery becomes a critical bottleneck in globally distributed primary biodiversity data infrastructures [11–13].

In contrast to term-level indexes, *full-text indexes* store a set of search keys that consist of all feasible substrings of the text. This is relevant for some Eastern languages (Burmese, Chinese, Taiwanese, Tibetan, etc.), which do not have a well-defined notion of words [14]. Full-text indexes are also required in Bioinformatics and Genetic applications, where finding the longest common subsequence problem (LCS) is a challenging issue [15,16]. In this field, a considerable number of DNA sequences have to be processed efficiently, both in space and time. However, the bottleneck is the space complexity of implementations because the structures often require more space than the original text [17].

Regarding term-level indexes, there are many data structures to implement the string dictionary, from Hash Tables to a variety of Trie representations, which try to overcome their compression rate handicaps. One Trie variation is the Double Trie (DT) proposed in [18], which segments the terms to index into two parts, where the left part is stored in a Trie of prefixes, and the right one in a Trie of suffixes. In this paper, we propose an extension of the DT that we have called MergedTrie, which achieves a higher compression by merging both Tries into a single, and by segmenting the term into two fixed length parts. Moreover, we propose a new implementation of Tries that achieves better compression rates than the double-array representation [19,20] usually chosen for implementing Tries.

Another application of Tries is the IP lookup for scalable virtual routers for network virtualization, which means running multiple virtual router instances in parallel [21]. Virtual routers have the scalability challenge, suffering from critical spatial efficiency issues when storing all the forwarding tables. Here, high-speed memory becomes a decisive issue. Moreover, security issues are especially crucial in these environments, which benefit from the Trie organization [22].

The paper is structured as follows. In section 2, we summarize the most relevant work related to our proposal. In section 3, we introduce our proposal. In section 4, we detail the implementation of the MergedTrie. In section 5, we experimentally analyze its efficiency. We conclude the paper with a summary of the main contributions and the directions for future work.

## 2.Related work

In the following subsections, we summarize: 1) the most relevant data structures for term-level indexing; 2) some implementation issues for these structures; 3) our contributions to the state of the art described in these subsections.

### 2.1. Data structures for term-level index

The *Trie*, also called the *Digital Tree* or *Prefix Tree* [23,24], is one of the main data structures used for indexing text. It is a tree for storing strings in which there is one node for every common prefix and the strings are stored in additional leaf nodes. Unlike a *Search Tree*, the nodes in the Trie do not represent elements stored in the structure. Instead, the merger of the characters of the nodes that form the path from the root to the node represent the stored string [25]. The Trie is a rather compact structure because overlapping prefixes are stored only once. It resolves a query that tests whether a given string belongs to the Trie in an amount of time which is proportional only to its length, unlike other data structures such as *Search Trees* or *Hash Tables* (an associative array whose keys are mapped to array positions by hash functions) in which the search time is a function of the number of elements stored. Therefore, Trie search operations are faster because in most dictionaries the number of words is higher than the maximum length of a word. Moreover, Tries do not require complex re-balancing or tree rotation operations. Furthermore, Tries can be used to represent full-text indexes too: for example, the *Suffix Trie* is a Trie that stores all the suffixes of a given string.

The main disadvantage of Tries is the high memory consumption, especially when the set of words is heterogeneous with a low degree of prefix sharing. This drawback is partly solved by its compact representation called *Radix Tree*, *Patricia Trie*, *Compact Prefix Tree* or *C-Trie*, where all nodes with one child are merged with their parents. In [26], a dynamic construction algorithm for a Compact Patricia Trie is proposed to solve the problem of the Patricia Tree requiring many good physical storage spaces in the memory, especially when the key set is too large to fit in its virtual memory.

In addition to the compaction operation, another operation to improve the memory consumption in Tries is minimization. The resulting structure is called *Directed Acyclic Word Graph (DAWG)* or *Acyclic Deterministic Finite Automata (ADFA)*, where unlike compaction, each edge between nodes is labelled with a character and consequently the number of nodes is significantly reduced. Essentially, a DAWG/ADFA is a finite state machine that recognizes a set of words [27]. When both compaction and minimization are performed, the *Compact Directed Acyclic Word Graphs (CDAWGs)* are obtained [28].

Due to compaction and minimization, the O(L) temporal efficiency of Tries, where L is the length of the string, is aggravated in insertions, updates and deletions in CDAWGs, DAWGs and Radix Trees. It occurs when compacted or minimized segments need to be split or merged, and a trade-off has to be made between the smaller size of the compressed Trie and the update speed. For example, in [29], an algorithm to create DAWGs is proposed, but the problem is that the words to insert must be arranged in alphabetical order, which introduces the difficulty of the sorting process as well as making it difficult to use them as a dynamic structure that allows future insertions and updates. It requires an additional Hash Table to represent the right languages to compact, which increases the time complexity of the whole algorithm to O(L log Q), where Q is the total number of states (nodes) in the (compacted) dictionary. Some authors have proposed algorithms to overcome these drawbacks. For example, in [30], a method to modify (adding and removal) a minimal finite-state automaton is proposed with a time complexity in the same class as in [29]. In [31], the author experimentally compares various methods for constructing DAWGs in an incremental, semi-incremental, and non-incremental way, from sorted and unsorted data. In this comparison, the impact of the intermediate structures required for the DAWG/ADFA construction is analyzed. In [32], the author presents a linear-time algorithm O(Q) for the minimization of acyclic deterministic finite-state automata (DAWG/ADFA). In [33], the author presents an algorithm that directly (i.e. without deleting any state) constructs pseudo-minimal DFA, without using a Trie-like DFA as an intermediate step, with a complexity of O(Q log(Q) + L). A taxonomy of the most important finite state minimization algorithms and a presentation of the known algorithms solving this problem, including some new ones, can be found in [34]. In [35], the authors show the relationship between the two most widely used approaches for the minimization of deterministic finite automata: minimization by splitting partitions and minimization by double reversal.

Another noteworthy work that highlights the necessity of both temporal and spatial efficiency is the Burst Trie proposed in [36], which focuses on the reduction of memory requirements as well as optimizing search operations. In the Burst Trie, each leaf holds a set of strings in a container (a container can be any data structure that is reasonably efficient for small sets of strings, such as a list or a binary search tree), allowing dramatic space reductions with no impact on efficiency. When the containers are full, they "burst" and are turned into branches. The authors experimentally proved that Burst Tries are faster than compact Tries by using just one-sixth of the space, and are close to hash tables in efficiency.

An interesting new data structure is proposed in [37]: INSTRUCT (INdexing STrings by Re-Using Common Triplets). Its remarkable advantage over previous work is that INSTRUCT efficiently handles both prefix and suffix search queries, as well as the exact string search operation. Moreover, it reduces the memory requirement by means of bit vectors for reusing the storage space for common triplets. The authors proved that INSTRUCT outperforms some existing structures by up to a factor of two in memory requirements while maintaining better or comparable running times for searching and insertion.

In conclusion, some structures achieve higher and more efficient data storage than Tries (e.g. Radix Tree, Patricia Trie, DAWG, ADFA and CDAWG) for term-level index, but they increase the complexity of insertion, update and removal operations, making it proportional to the number of nodes/states (Q), as well as requiring intermediate structures to perform the minimization/compaction process. As a way to balance temporal and spatial efficiency, the Double Trie (DT) was proposed in [18,38], and implemented by Watson [39] in C++ and in Java in the toolkit known as FIRE Engine II (http://www3.cs.stonybrook.edu/~algorith/implement/watson/implement.shtml). It compacts the Trie by segmenting the terms to index into two parts, where the left part is stored in a Trie of prefixes, the right one in a Trie of suffixes, and both Tries (and both parts of the term) are linked. Thus, a compact process is performed automatically, which obtains structures almost as small as the ADFA, but which can be constructed much faster.

### 2.2. Implementation of data structures for the term-level index

Other authors focus their efforts on improving space consumption by means of sequential implementations, instead of pointer representations. For example, the CMU-Cambridge language modelling toolkit [40,41] represents the Trie as contiguous arrays of fixed-size node records, where each array corresponds to a certain layer of the Trie. In addition, [19,42] employ a *double-array* structure for the Trie, formed by three one-dimensional arrays to represent a reduced Trie: the array BC to represent Trie nodes through two integers, the array TAIL to store suffix strings, and an additional array to indicate character codes. However, their comparative evaluation with the list representation for Tries shows that their proposal is slower for the insertion operation, and concludes that their structure should be improved for large sets of keys. Several authors have tried to overcome these drawbacks, such as [43] who tried to speed up the insertions, [44] who tried to eliminate efficiently the empty elements, [45] who tried to compact the double array structure, and [46] who tried to divide the Trie into multiple levels and to remove the BASE array.

Given that our proposal extends Double Tries and improves on the double-array implementation, the work in [47,48] is especially interesting to analyze. They propose a new double-array representation with string labels using multiple arrays depending on label sizes, used to implement the Double Trie and the Minimal-Prefix Trie (only the minimal prefixes of keywords are kept as Trie nodes, and the suffixes are kept separately as strings). In our experimental evaluation section, we will compare our proposal with those mentioned in the work by Kanda et al.

The work of [8] address Trie compaction through Tightly Packed Tries (TPTs), a compact implementation of read-only, compressed Trie structures with fast on-demand paging and short load times. They represent the Trie in a single contiguous byte array, after traversing it in post-order. For each node, they store the byte offsets of its children, the node value, the size of the index, and the actual index as a list of alternating token IDs and byte offsets. The main drawback of this proposal is that it does not allow for the updating of the TPT (i.e., it is a read-only data structure).

More variants of Tries can be listed. For example, HAT Tries [49] are a type of Radix Trie using array nodes to collect key-value pairs under radix nodes and hash buckets into an associative array. Other examples are the *Hash Trie* and the *Hash Array Mapped Trie* (HAMT) [50]. The Hash Trie is a persistent data structure that can be used to implement sets and maps. In its basic form, a Hash Trie stores the hashes of its keys, regarded as arrays of bits, in a Trie, with the actual keys and (optional) values stored in the Trie’s final nodes. The HAMT is based on the notion of hashing a key and storing it in a Trie based on this hash value.

Currently, Tries are also used in IP lookup for scalable virtual routers for network virtualization, to run multiple virtual router instances in parallel on a common physical platform [21]. Virtual routers have the scalability challenge, which requires a significant amount of memory to store all the forwarding tables. Here, high-speed memory becomes a critical issue. As the authors state, IP lookup solutions fall into three main categories: ternary content addressable memory (TCAM)-based, Hash-based, and Trie-based solutions. The first suffers from the limitations of excessive power consumption, high cost, and low density. The Hash-based solution requires prohibitive amounts of high-bandwidth memory that prevents their use in practice. The Trie-based solution improves upon the previous ones by performing the longest prefix matching (LPM) operations. The proposal by [21] focuses on merging multiple separate Tries into a shared data structure in order to achieve significant memory reductions for a large number of forwarding tables in virtual routers, but the complexity of insertion and modification operations is increased. The work by [51] concludes that this kind of Trie overlay works well when the separate forwarding tables have similar structures. Otherwise, simple overlaying cannot lead to any memory reduction, even causing a significant increase in memory usage. *Trie braiding* [52] is an alternative approach, which is designed to increase the overlap among multiple forwarding tables by using a child rotation mechanism. However, the authors conclude that the reduction in the total number of nodes does not necessarily lead to a reduction in overall memory usage. This is because no memory is allocated to leaf nodes, and non-leaf nodes dominate the memory consumption of the Trie data structure.

In conclusion, previous works report the sequential implementation of these data structures by means of one or more arrays (e.g. a double-array structure), reaching a high level of compaction. Moreover, some approaches use intermediate structures such as Hash Tables in order to improve the running time, although they increase memory usage. Finally, it must be highlighted that some of these implementations achieve high compression, but they construct read-only static dictionaries (i.e. they do not support updates).

### 2.3. MergedTrie contributions to the state of the art

In this subsection, we highlight the theoretical and practical impact of our research, a data structure for dynamic term-level index (keyword search) operations in string dictionaries, which is necessary in applications such as Information Retrieval, social networks and IP lookup.

In terms of the theoretical impact of our research, our proposal is called MergedTrie because in a single Trie it merges the two Tries used in the Double Trie (DT) [18,38] in order to achieve both prefix and suffix overlapping in the terms to be indexed. Moreover, the MergedTrie enhances the DT segmentation of the term in order to achieve a reduction in the height of each Trie. We segment the term in a fixed way, at exactly half of the term’s length, whereas the DT segments the term in a flexible way according to the insert process (when the search in the prefix Trie fails, the remaining string is added to the suffix Trie). Therefore, the MergedTrie improves memory consumption by significantly reducing the number of states/nodes in DT, as DAWG/ADFA do, but overcoming the problem of the significant cost of the update operations (e.g. when compacted or minimized segments need to be split or merged). For that reason, the MergedTrie achieves optimal spatial efficiency as well as high temporal efficiency, outperforming even the Hash Table (unlike the Burst Tries by [36]). Consequently, the MergedTrie supports term-level index operations such as keyword insertion, search, modification, deletion, listing and prefix listing. Similarly to the INSTRUCT structure by [37], the MergedTrie also supports suffix listing since the suffixes are also stored in a Trie fashion, but we outperform more than two factors in memory requirements over some structures, as the experimental evaluation section will show. Moreover, it does not need to perform a compression/decompression process (e.g. [6,53]), nor does it require a pre-sorting process on the set of words to insert (e.g. [29]). The MergedTrie involves quite simple updating operations based on Trie operations, resulting in a complexity in the insertion, search, modification and deletion operations that is proportional to the length of the string (DAWG/ADFA reports an O(Q) complexity, where Q is the number of states/nodes). Finally, the MergedTrie facilitates the storing of auxiliary (e.g. a dictionary of geographical names with their GPS information associated) and additional information (e.g. n-grams, phrases, bitexts or biwords) about each word, because it automatically provides word identifiers that different MergedTries can share, which is more difficult for ADFA terminal nodes that can be reached through multiple paths.

Concerning the practical impact of our proposal, we have proposed a new implementation of Tries and MergedTries in C++ by means of a sequential representation that also allows for efficient storage in secondary memory. This way, the subsequent load times are optimized and direct access to the structure is permitted when the MergedTrie does not fit in the main memory due to the number of keys to be indexed [26]. Thus, the MergedTrie becomes a completely dynamic and persistent structure. Moreover, it has been experimentally compared with the Hash Table, Trie, Double Trie, double array, Crit-bit and ADFA-DAWG (implemented in order to allow unsorted term insertion, and another implementations such as a double-array structure) to prove its theoretical benefits, achieving the aim of requiring less space than the original text to be indexed, overcoming the space bottleneck complexity caused by structures which often require more space than the original text [17].

## 3. The proposed data structure: MergedTrie

**Fig 1:**
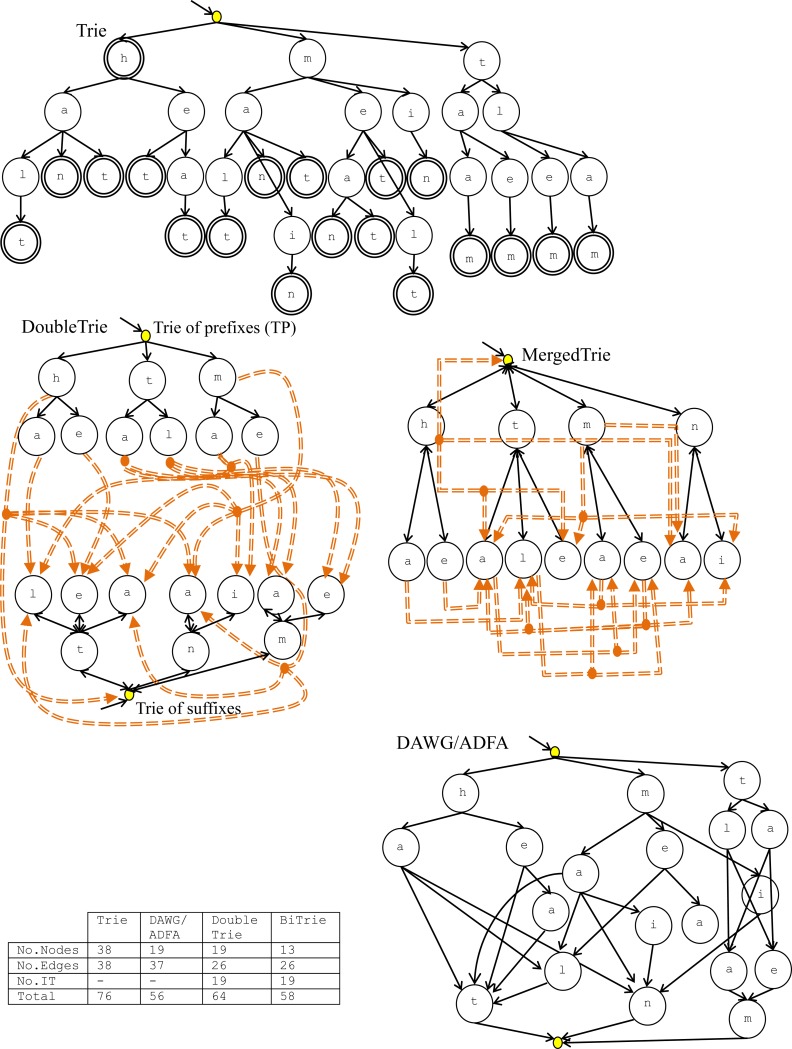
Trie, Double Trie, MergedTrie, and DAWG/ADFA formed by 19 words: h, hat, halt, han, heat, het, main, malt, man, mat, met, meat, mean, melt, min, taam, taem, tlam, tlem.

Next, the MergedTrie structure is explained in full, with the extensions to the Double Trie analyzed in the previous subsection. It consists of the linking of two Trie structures: one for storing the prefix (from now on: the *Trie of prefixes* or TP) and the other for storing the suffix (from now on: the *Trie of suffixes* or TS) of each word. The prefix and suffix of each word are obtained from the segmentation of the word into two parts of equal length (e.g. the term *halt is* segmented into *ha* and *lt*). The TS stores each suffix in its reverse order (i.e. the suffix *lt* is reversed into the string *tl* so the character *t* is stored in the root of the TS). The last node of the prefix in the TP will link to the last node of the TS (i.e. the first character of the suffix, since the suffix is stored in its reverse order) by means of a special type of edge called an *inter-trie* (IT). In MergedTries, both TP and TS are merged into a single Trie (T) that supports descending and ascending traversal. In Fig 1, an example of a MergedTrie is shown, as well as its equivalent Double Trie (with two Tries), Trie and DAWG/ADFA. In this Figure, the IT links are depicted as orange double-lines, which replace the “end of word” flags, represented in the Trie as double-lined nodes. In this way, each IT link marks a term indexed in the MergedTrie, which is also used to store the auxiliary information about each word if required (e.g. a dictionary of a company’s staff with their personal information associated). Moreover, a prefix can have several suffixes such as *ma* with *lt* and *in*, for the terms *main* and *malt* respectively (this fact is shown in Fig 1 with orange dots). In the case of words formed by one character (e.g. the word *h*), the character is stored in T with an IT link to the root.

1. *Insertion*(string w; MergedTrie b) {
2.         string wPrefix, wSuffix;
3.         TrieNode *lastPrefixNode, *lastSuffixNode;
4.         (wPrefix, wSuffix) = StringSegmentation(w);
5.         lastPrefixNode = b.Trie.Insertion(wPrefix);
6.         lastSuffixNode = b.Trie.Insertion(wSuffix.ReverseOrder);
7.         lastPrefixNode->CreateIT_Node(lastSuffixNode);
8. }
9. bool *Search*(string w; MergedTrie b) {
10.        string wPrefix, wSuffix;
11.        TrieNode *lastPrefixNode, *lastSuffixNode;
12.         (wPrefix, wSuffix) = StringSegmentation(w);
13.         lastPrefixNode = b.Trie.Search(wPrefix);
14.         if(lastPrefixNode->exist()) {
15.                 if(lastPrefixNode->IT_exist()) {
16.                          if(w.length() = = 1) {
17.                                   if(lastPrefixNode->IT_root_TS_exist())
18.                                            return true;
19.                                   else
20.                                            return false;
21.                          } else {
22.                                   lastSuffixNode = b.Trie.Search(wSuffix.ReverseOrder);
23.                                   if(lastSuffixNode->exist()) {
24.                                       if(exist_IT(lastPrefixNode, lastSuffixNode))
25.                                                  return true;
26.                                       else
27.                                                  return false;
28.                               } else
29.                                       return false;
30.                        }
31.               else
32.                        return false;
33.       } else
34.               return false;
35. }

**Algorithm 1**. Insertion and Search procedures in MergedTrie.

The insertion algorithm is presented in Algorithm 1, which shows its simplicity. It is based on the segmentation (*StringSegmentation* in line 4) of the word to insert (*w*) into its prefix and suffix (*wPrefix*, *wSuffix*). When the length of *w* is odd, the suffix is one character longer than the prefix (e.g. *hat is* segmented into *h and at*). This excludes the words formed by one character (e.g. the word *h*), where the character is stored with an IT link to the root. Afterwards, *wPrefix* is stored in T (line 5), whereas *wSuffix* is stored in T in its reverse order (line 6). As previously described, the IT links the *lastPrefixNode* to the *lastSuffixNode* (line 7). Therefore, the MergedTries insertions exhibit O(L) temporal complexity (where L is the length of the searched term), because it is reduced to prefix and reverse order suffix insertions in simple Tries, as well as adding the IT link between their prefix and suffix.

The term search operation is presented in line 9 in Algorithm 1, which is quite similar to the insertion procedure. It segments (line 12) the word to search (*w*) into its prefix and suffix (*wPrefix*, *wSuffix*) as described for the insertions. The prefix is searched in T (line 13). If the prefix successfully exists in T (line 14), then lastPrefixNode must have at least one IT link in order to address the first character of *wSuffix* (line 15). Otherwise, *w* is not in the MergedTrie (line 32 and 34). In the case of terms formed by just one character (line 16), an IT link to the root of T must exist (line 17), in which case *w* is in the MergedTrie (line 18); otherwise, *w* is not there (line 20).

Afterwards, *wSuffix* is searched in reverse order in T (line 22). If *wSuffix* successfully exists (line 23), then an IT link that joins the last prefix node with the last suffix node must exist (line 24). Otherwise, the MergedTrie does not contain *w* (line 27 and 29).

For example, *mein* is not found in the MergedTrie in Fig 1 because line 15 concludes that no IT link exists between nodes *e* and *i*. Line 24 is required for cases in which several IT links involve the same character. For example, let us suppose that in the MergedTrie in Fig 1 the term *meat* is not stored. When *meat* is searched, line 15 would succeed because the term *mean* exits, but line 24 would not because the proper *a* node would not be IT linked from the *e* node.

The search operation in MergedTrie also shows a temporal complexity of O(L).

That is because the search operation simply requires traversing the prefix and suffix Tries. Moreover, the MergedTrie improves the temporal efficiency of Trie searches in cases such as the search of *heatwave* in the Trie in Fig 1, in which after traversing part of the prefix (*he*), line 14 concludes that the term does not exist although the string *heat* appears in the Trie. Similarly, the efficiency is improved by line 15, when the last node of a string in the Trie of prefixes does not contain an IT link.

The deletion operation also presents O(L) temporal efficiency because it consists of a search operation and the subsequent IT link deletion. Regarding nodes to be deleted that are not used for other terms in the MergedTrie, they are deleted by a new operation called “compaction”, which has been designed with the aim of improving the MergedTrie computational efficiency in the implementation that is detailed in section 4.2.

The prefix listing is performed with a similar complexity to Tries because it consists of the traversing of each IT link in the TP path. For each IT link found in this path, the nodes in the TS are traversed in ascending order. That is why in Fig 1, the links between the nodes of T are two-way arrows in order to allow for ascending traversal. For example, the ha* prefix listing in Fig 1 is performed by traversing the IT links of the *h* and *h+a* nodes of T. The former reaches three IT links to the words *het*, *hat* and *han*. The first is discarded because it does not satisfy the prefix requirement, but the remaining terms are listed. The latter reaches the word *halt* that is also listed. In case of prefix listings longer than the one stored in the TP, the operation works in a similar way, because the maximum string length in T is traversed, and all the IT links of the last prefix node are traversed and checked to see if they satisfy the prefix requirement. For example, the mea* prefix listing in Fig 1 is performed by traversing the IT links of the maximum length, the *m+e* node, which has three IT links to the words *mean*, *meat* and *melt*. Here, the last word is discarded.

In conclusion, the table in Fig 1 summarizes the comparison between the Trie, Double Trie, MergedTrie and DAWG/ADFA, all indexing the same set of 19 words. As can be observed, the Trie is quite inefficient regarding the number of nodes and edges (76 in total) in comparison with the reduction to 56 (DAWG/ADFA), 58 (MergedTrie) and 64 (Double Trie). Thus, these figures show the improvement of the MergedTrie over the Double Trie (58 vs. 64). The DAWG/ADFA is slightly more efficient than the MergedTrie because the number of edges (two-way arrows vs. one-way in DAWG) and IT (one for each term vs. zero in DAWG) is higher, although the number of nodes is lower (13 vs. 19 in DAWG). This small increase is offset by the great simplicity of the insertion and updating operations, with a much better complexity proportional to L, compared to Q (the total number of states/nodes), as the experimental evaluation section will prove.

## 4. MergedTrie implementation

This section details the implementation and is divided into two subsections: 1) the C++ sequential implementation of the MergedTrie; 2) the improvement in its computational efficiency by means of the compaction operation.

### 4.1. The sequential implementation of the MergedTrie

This section contains details of the MergedTrie implementation which will be used in the comparative experimental analysis in section 5. We have implemented the MergedTrie in C++, an efficient programming language for the proper processing of memory, and frequently used in text applications (e.g. in Information Retrieval). The MergedTrie is represented using one-dimensional arrays, unlike the traditional pointer-linked structure of Radix Trees and Tries: memory pointers are replaced by offsets for the links between nodes. This is especially important on 64-bit machines, where pointers require 8 bytes. Thanks to this sequential representation, after the MergedTrie is created, when it is stored in secondary memory, the subsequent load times are significantly reduced because the dynamic reservation of the array memory is performed in one single operation. Loading each array position is more efficient than the link representation, which means creating and linking each node individually. Moreover, it allows direct access to the structure when it is stored in secondary memory if it does not fit in the main memory due to the number of keys to be indexed. Therefore, thanks to the one-dimension array representation, the de/serialization of the MergedTrie from/into the disk is quite simple and efficient.

**Fig 2:**
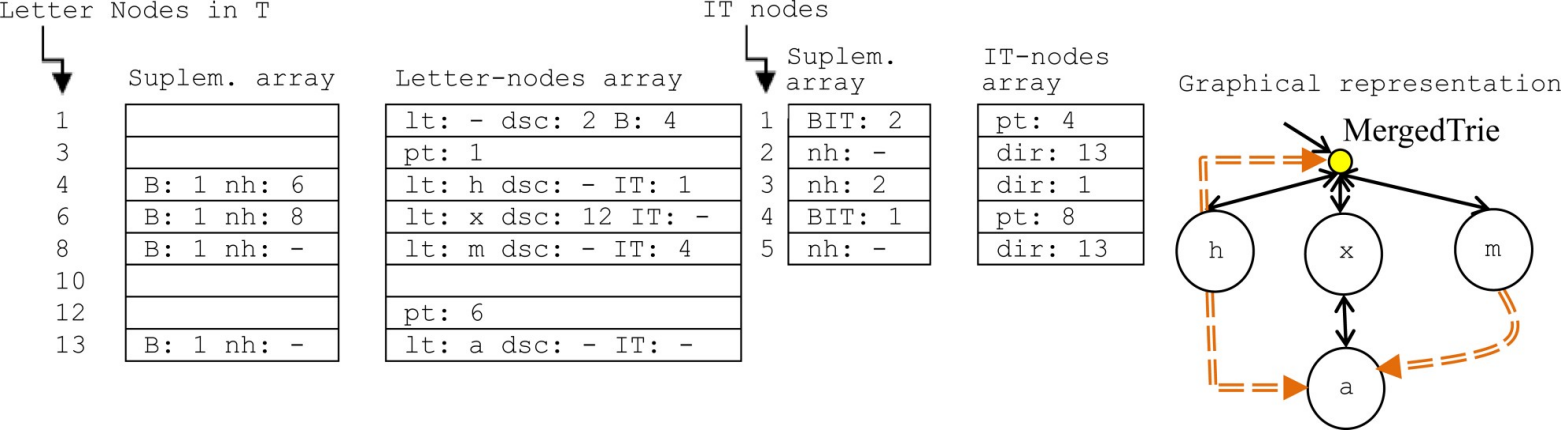
Sequential and graphical representations of the MergedTrie with the words *h*, *hax*, *max* (*lt*: letter; *dsc*: descendant index; *IT*: inter-trie index; *pt*: index of the parent node; *nh*: index of the following hash collision; *dir*: IT index to suffix letter-node; *B*: size of hash table of letter-nodes; *BIT*: size of hash table of IT links; root of T: 1).

Our structure remains totally dynamic, increasing or decreasing the array dimension if this is required. Specifically, we are using one array for the letter-nodes in the Trie (T), and another for the inter-Trie nodes (IT), both “unsigned int” (32 bits). Moreover, two additional “unsigned char” (8 bits) arrays are used for supplementary information. Each attribute of the MergedTrie nodes is stored in these arrays by C++ bit fields as detailed in Table 1, where the letter-nodes are stored in 72 bits (two sequential positions in the letter-node array plus a position in its supplementary array), and the IT-nodes in 40 bits (one position in the IT-node array plus a position in its supplementary array). The *parent/moved/IT/BIT bit* fields are used to differentiate between different kinds of nodes in the arrays, and the remaining fields/attributes are depicted in the MergedTrie with the words *h*, *hax* and *max* in Fig 2. In its sequential implementation, the root of T is stored in index 1. All letter-node descendants or IT-nodes of a letter-node are represented as Hash Tables of sizes B and BIT respectively, and collisions between nodes are solved by searching for an empty bucket (e.g. bucket 10 in the letter-nodes array Fig 2) in the Hash Table. Collisions are linked by means of lists through the *nh* field (e.g. bucket 4 in the letter-nodes supplementary array presents *nh*: *6*, which means that the next colliding letter-node is the one in position 6). For example, the Hash Table of descendant letter-nodes of the root of T (see index 1 in Fig 2) is of size B = 4 and starts in index *dsc*: *2*, so it is formed by bucket 2 (*pt*: *1* that means that the parent node is in index 1, in order to support the ascending traversals of T) and buckets 4 to 10 for the Hash Table (each letter-node is stored in two sequential positions of the array).

**Table 1:** C++ bit fields used in the MergedTrie implementation.

Letter-node		IT-node	
Field	Num. bits	Field	Num. bits
Parent Node bit	1	IT vs. BIT node bit	1
Moved/Letter Node bit	1	BIT/offset of the following hash collision	13
Letter	8	Index to suffix letter-node/index to prefix letter-node	26
Descendant offset	24		
Moved offset/Parent offset/IT index	30		
B/offset of the following hash collision	8		
TOTAL	72	TOTAL	40

If a Hash Table is full, B is increased and the Table is created in a new array position, in which the letter-nodes in the full Table are replicated in the new Table by means of *moved nodes*, which only have the offset to the original letter-node. In this way, the previous IT addresses are not affected by the re-dimensioning operation.

The IT links are represented in the letter-nodes when the *IT* field is different to ‘–‘, such as the *h* letter node in bucket 4, whose IT links are stored in the Hash Table that starts in index *IT*: *1*. Bucket 1 contains the size of the IT hash table (*BIT*: *2*), and the following two BIT positions compose the proper Hash Table. In this case, it is comprised of two IT links to the letter-nodes in T: *dir*: *13* (letter-node *a*, so it marks the end of word *hat*) and *dir*: *1* (root node, so it marks the end of word *h*). Both IT links are synonyms, so the collision is solved by the link *nh*: *2* (see bucket 3 in the IT-nodes supplementary array).

Each term is identified unambiguously by one IT-node that will carry the data associated with the term, which is stored separately (e.g. a *MergedTrie<float>* will store the set of float values associated with each term stored, such as *<computer*, *1*.*3>*). This feature provides an additional contribution to represent relationships between different words, as it is required in n-grams, phrases, cryptographic codes, bitexts or biwords. Other authors (e.g. [54–56]) store these resources by assigning codes to words. The MergedTrie automatically provides these codes or word identifiers: the array index of IT links. For example, in Fig 2 the following word identifiers are automatically assigned to the words: *h (*3), *hax* (2) and *max* (5). Therefore, we can easily store the bi-gram frequency of the pair “*hat-mat”* by indexing the identifiers “2–5” in an additional data structure.

### 4.2. The compaction operation

This optional operation can be run on-demand with the aim of improving the temporal and spatial efficiency of the MergedTrie. It runs a level-order (also known as breadth-first) traversal of the MergedTrie, and gathers the letter nodes that share sibling or descendant relations, storing them in close array positions. In this way, after the compaction operation, the descendant offsets are fewer than those in the non-compacted MergedTrie, as well as making our implementation cache-friendly. In addition, the moved or deleted nodes are also removed.

**Fig 3:**
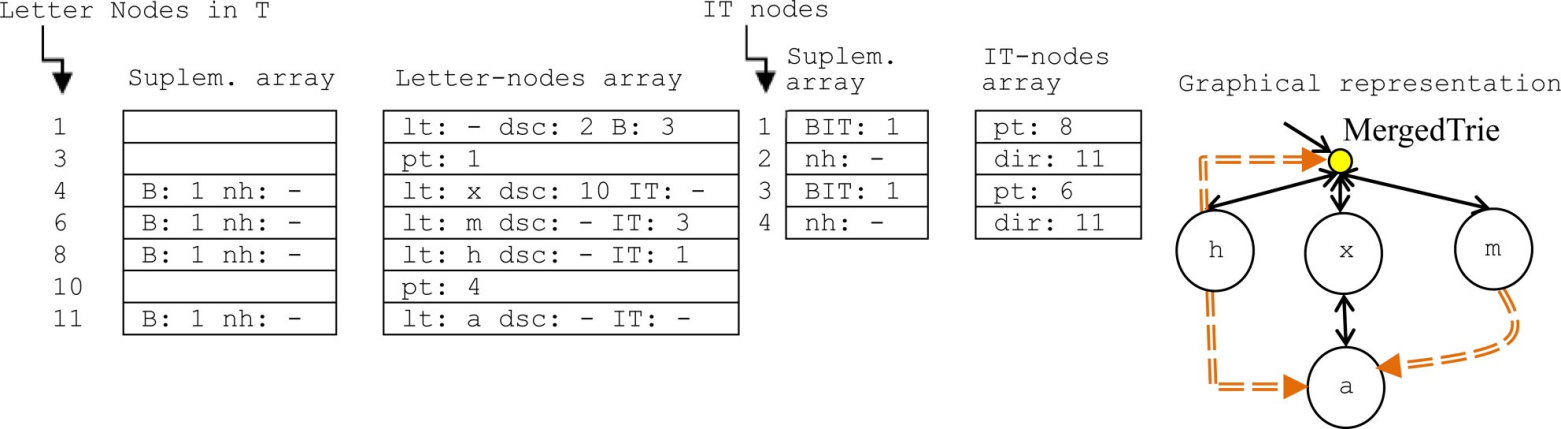
Sequential and graphical representations of the MergedTrie in Fig 2 after deleting the word *h* and running the compaction operation. Words *hax* and *max*. (*lt*: letter; *dsc*: descendant index; *IT*: inter-trie index; *pt*: index of the parent node; *nh*: index of the following hash collision; *dir*: IT index to suffix letter-node; *B*: size of hash table of letter-nodes; *BIT*: size of hash table of IT links; root of T: 1).

Moreover, in the compaction operation, the size of the hash tables (*B* and *BIT*) is properly adjusted according to the number of items that they contain in order to avoid empty buckets. For example, in Fig 3, the MergedTrie in Fig 2 is depicted after deleting the word *h* and running the compaction operation. The letter-node in bucket 1 in Fig 2 with *B = 4* is set to *B = 3* in Fig 3 in order to clear the empty bucket number 10 in Fig 2. This adjustment of *B/BIT* forces the re-hashing of the items, which reduces the length of *nh* lists, and eradicates collisions between no-synonym items. For instance, in Fig 2, the *x* character is a synonym of *h* that already occupies the bucket 4 because *x MOD 4 = h MOD 4 = 0*, hence *x* is stored in bucket 6. However, when *m* has to be stored in Fig 2 in bucket 6 (m MOD 4 = 1), it is occupied by the *x* collided item that is not a synonym of *m*, which forces us to search for an alternative position and to link it properly by *nh* (bucket 8). The re-hashing of *h*, *x* and *m* with B = 3 achieves an optimal distribution as Fig 3 shows: x MOD 3 = 0; m MOD 3 = 1; h MOD 3 = 2. Therefore, the temporal efficiency is improved because the previous two collisions are eliminated. Moreover, the deletion of word *h* means that the IT bucket 3 is removed and the BIT in bucket 1 is set to 1. As can be observed in Fig 3, the spatial efficiency is improved after compaction because three buckets are removed.

## 5. Experimental evaluation

Two experiments have been performed to prove the benefits of our proposal, which are analyzed in the following two subsections. The first experiment measures the benefits in the spatial efficiency of the MergedTrie, whereas the second one focuses on the temporal efficiency analysis.

These experiments have been run on a 64-bit 8 x Intel(R) Xeon(R) CPU E5606–2.13GHz—32 Gb RAM computer with Ubuntu14.04 LTS (Linux 3.13.0-95-generic SMP x86_64 GNU/Linux). Our C++ implementation has been compiled with GCC version 4.8.4 with the options “-std = gnu++0x -O3”.

Two different datasets have been used in the experiments. The first one (TREC+) is formed by 7,184,348 different words with a size of 3.2 GB. It is the result of merging the following files: five English collections, mainly about newspapers; documents (http://trec.nist.gov/data/docs_eng.html) used in TREC-8, 9 and 10; Question Answering competitions; a dictionary of Spanish [57]; and the dictionaries reported in the Apertium project (https://sourceforge.net/p/apertium/svn/HEAD/tree/trunk/). The second one (Google1Grams) is formed of 58,654,411 different words from the joining of the English, Chinese, French, German, Hebrew, Italian, Russian and Spanish Google 1-grams (http://storage.googleapis.com/books/ngrams/books/datasetsv2.html) in UTF-8 codification. In this way, we prove the benefits of our proposal in several sized corpora.

The MergedTrie will be compared to several well-known index structures implemented in C++: (1) our Trie implementation as a pointer-linked structure; (2) the STL Hash Table “unordered_set<string>”; (3) the binary Crit-bit (also known as PATRICIA) tree structure (Adam Langley, https://www.imperialviolet.org/binary/critbit.pdf); two implementations of DAWG: (4) the one by Susumu Yata by means of a double array as a base structure (dawgdic-0.4.5 https://code.google.com/archive/p/dawgdic/downloads), which requires insertions of sorted text; (5) the one used in [31] in http://www.jandaciuk.pl/adfa.html, which allows insertions of unsorted terms; and [47,48] implementations: (6) double-array Trie; (7) double-array Trie with string labels; (8) double-array Minimal-Prefix (MP) Trie; (9) double-array MP Trie with string labels; (10) Double Trie implemented as a double-array; (11) Double Trie with string labels implemented as a double-array. These structures will show an external evaluation and comparison to current, efficient and widely used data structures.

### 5.1. Experiment 1: Spatial efficiency

The memory consumption shown in Table 2 has been measured using a C++ program (http://dis.um.es/~ginesgm/files/doc/memory.cpp; http://dis.um.es/~ginesgm/medidas.html#mt) that returns the maximum memory usage in kilobytes by scanning the file “/proc/process_id/smaps” 16 times per second. The percentages (Δ %) mean the rate of variation in the maximum memory usage with regard to the “(0) MergedTrie”.

**Table 2:** Max. memory usage (kB) with both corpora: TREC+ (7,184,348 words) and Google1Grams (58,654,411 words).

Data structures for term-level index	Corpus TREC+		Corpus Google1Grams	
	Max. memory usage(kB)	Δ %	Max. memory usage(kB)	Δ %
(0) MergedTrie	56,876		524,234	
(1) Trie	686,680	1,107.3%	12,850,236	2351.2%
(2) Hash Table	637,360	1,020.6%	6,653,784	1169.2%
(3) Crit-bit	449,344	690.0%	3,740,840	613.6%
(4) DAWG-Yata as a double array	340,196	498.1%	2,888,212	450.9%
(5) DAWG-Daciuk	185,668	226.4%	4,335,440	727.0%
(6) Double-array Trie	104,128	83.1%	1,209,780	130.8%
(7) Double-array Trie with string labels	81,851	43.9%	740,333	41.2%
(8) Double-array MP Trie	70,223	23.5%	706,482	34.8%
(9) Double-array MP Trie with string labels	67,607	18.9%	637,301	21.6%
(10) Double Trie as a double-array	62,427	9.8%	581,382	10.9%
(11) Double Trie as a double-array with string labels	61,526	8.2%	539,039	2.8%

As Table 2 shows, the MergedTrie is the structure that obtains the best spatial efficiency results. It reaches a maximum variation from 56,876 kB (MergedTrie) to 686,680 kB (Trie), which is the structure that requires the most memory. As expected, the Hash Table is also quite memory inefficient (637,360 kB). The second most efficient structure is the (11) Double Trie as a double-array with string labels proposed in [47,48], although it increases its memory consumption by 8.2%, even when Kanda et al. structures achieve a high compression rate given that they implement static dictionaries (i.e. that do not support updating operations as the MergedTrie does). These results are confirmed for the Google1Grams corpora.

From these results, we can conclude that the MergedTrie clearly outperforms up-to-date structures specialized in term-level index, showing a high reduction of memory even when the MergedTrie supports insertions, updates and removals of terms (i.e. it is not a static dictionary).

Regarding the comparative analysis of the number of nodes/edges, the results reported by the structures that we have implemented are the following in the format “number of nodes + number of edges = total”: (0) MergedTrie (our proposal with one Trie) (1,547,419 + 7,936,434 = 9,483,853); MergedTrie, as in Double Tries, with two Tries (1,609,816 + 7,967,202 = 9,577,018); (1) Trie (14,643,216 + 14,643,216 = 29,286,432). From these results, as expected, a high reduction in the number of nodes and edges is obtained by the MergedTrie with regard to the Trie: 9,483,853 vs. 29,286,432. The improvement in comparison to the MergedTrie with two Tries is also verified: 9,483,853 vs. 9,577,018, which proves the benefits of our proposals.

Regarding the contributions of the compaction operation (section 4.2), the number of positions occupied in the arrays is decreased from 28,212,579 to 11,589,966. Moreover, the number of collided nodes in the Hash Tables is decreased from 6,792,968 to 2,756,650.

### 5.2. Experiment 2: Temporal efficiency

The second experiment focuses on the temporal efficiency analysis. The running time shown in Table 3 has been measured in seconds by the *getrusage* function that reports resource usage totals for processes. The running time of the insertion and search for each term in the File is measured in the first two columns. The third column shows the efficiency of the search operation when the term to be searched is not in the File. This failure search is performed for the terms in the File when a random character is inserted in a random position in the original term. Each column result shows the time measured in seconds (s) and the percentage variation (Δ % where the negative values mean a percentage increase in comparison to the MergedTrie).

**Table 3:** Times in seconds in experiment 2 with TREC+ corpora (7,184,348 words).

	Insertion (s) Δ %		Success. search (s) Δ %		Failure search (s) Δ %	
(0) MergedTrie	5.51		2.57		1.98	
(1) Trie	7.15	29.6%	8.49	229.9%	7.16	261.2%
(2) Hash Table	6.14	11.4%	2.77	7.7%	3.75	89.0%
(3) Crit-bit	9.27	68.1%	7.61	195.8%	6.28	216.6%
(4) DAWG-Yata as a double array	12.52	127.1%	1.00	-61.2%	1.35	-31.7%
(5) DAWG-Daciuk	141.22	2461.4	-	-	-	-
(6) Double-array Trie	12.84	132.9%	0.78	-69.7%	1.11	-44.1%
(7) Double-array Trie with string labels	13.68	148.0%	1.21	-53.0%	1.35	-32.0%
(8) Double-array MP Trie	13.83	150.8%	0.88	-65.8%	1.13	-43.1%
(9) Double-array MP Trie with string labels	16.87	206.0%	1.22	-52.5%	1.34	-32.7%
(10) Double Trie as a double-array	15.60	183.0%	0.92	-64.3%	1.14	-42.8%
(11) Double Trie as a double-array with string labels	18.55	236.4%	1.31	-49.0%	1.37	-31.0%

As can be observed in Table 3, regarding the insertion operation, the MergedTrie outperforms the remaining structures for the insertion operation as was theoretically expected. The MergedTrie (5.51 s) especially outperforms the DAWG-Daciuk (141.22 s) that inserts unsorted terms, which proves the greater complexity of its insertion algorithm. The insertion time of the double array and the Double Trie static structures is much higher than the MergedTrie, with the additional advantage that the MergedTrie allows updates. The second fastest structure is the Hash Table (6.14 s), although as was shown in the previous experiment, it is quite memory inefficient.

Regarding the search operations, the DAWG-Daciuk structure is not analyzed because the software package provided by Daciuk does not accomplish these operations. With regard to the successful search operation, the DAWG and the structures implemented by [47,48] outperform the MergedTrie, but the MergedTrie outperforms the Trie, Crit-bit and Hash Table structures. However, it should be kept in mind that they require previous sorting of the terms to index and do not allow modifications of the indexed terms. After analyzing these results, we consider that the point of improvement in the proposed MergedTrie implementation is the number and length of the lists of collided terms, with a maximum of 7 letter-nodes and 11 IT-nodes. These figures mean that in the experiment, the searches for all the words in the File have to traverse each of these collided items, which results in a longer response time. Similar results have been obtained for the Google1Grams corpora (see Table 4).

**Table 4:** Times in seconds in experiment 2 with Google1Grams corpora (58,654,411 words).

	Insertion (s) Δ %		Success. search (s) Δ %		Failure search (s) Δ %	
(0) MergedTrie	37.15		27.36		16.16	
(1) Trie	243.30	554.9%	239.51	775.5%	183.37	1034.5%
(2) Hash Table	53.00	42.7%	30.00	9.7%	39.00	141.3%
(3) Crit-bit	87.93	136.7%	66.18	141.9%	55.75	244.9%
(4) DAWG-Yata as a double array	184.67	397.1%	8.57	-68.7%	10.42	-35.5%
(5) DAWG-Daciuk	2.283.29	6046.2%	-	-	-	-
(6) Double-array Trie	126.97	241.8%	6.54	-76.1%	11.87	-26.6%
(7) Double-array Trie with string labels	133.17	258.5%	10.66	-61.0%	14.41	-10.9%
(8) Double-array MP Trie	122.95	230.9%	7.19	-73.7%	12.01	-25.7%
(9) Double-array MP Trie with string labels	164.17	341.9%	10.75	-60.7%	14.29	-11.6%
(10) Double Trie as a double-array	129.41	248.3%	7.25	-73.5%	11.88	-26.5%
(11) Double Trie as a double-array with string labels	170.71	359.5%	11.76	-57.0%	14.51	-10.2%

Regarding the compaction operation (section 4.2), it is run in 1.94 s. The successful and failure search time is reduced by 15.6% and 8.7% respectively, which proves the benefits of eradicating the collisions between no-synonym items. Specifically, the number of collided nodes in the Hash Table is decreased from 6,792,968 to 2,756,650; and the maximum length of the lists of collided terms is decreased from 24 to 7 letter-nodes and from 1,227 to 11 IT-nodes.

## 6. Conclusions and future research

In this paper, we have proposed the MergedTrie data structure for term-level index operations, such as keyword insertion, search, modification, deletion, listing and prefix listing, necessary in applications that require string processing, such as Information Retrieval, social networks and IP lookup, especially crucial for Big Data and data mining applications (e.g. the biomedical text).

The main contributions of our proposal have been summarized according to the theoretical impact of our research as follows:

1. The MergedTrie extends the Double Trie (DT) structure [18,38] in the following ways:
  - It enhances the DT segmentation of the term in order to achieve a reduction in the height of the Trie by segmenting the term at exactly half its length.
  - It merges the two Tries in DT into a single one, in order to achieve both prefix and suffix overlapping in the terms to be indexed. Thus, it achieves a great decrease in the number of nodes and memory consumption.
2. It results in a great reduction in memory consumption, both in states/nodes and edges and in the prefixes and suffixes of the indexed terms, overcoming the problem of the significant cost of update operations as occurs when compacted or minimized segments need to be split or merged in the DAWG, ADFA and CDAWG structures.
3. It does not require prior term sorting, compression or decompression processes, as occurs in other “compact” structures or representations.
4. It keeps the complexity of the insertion, search, modification and deletion operations proportional to the length of the string, in contrast to DAWG/ADFA structures, where this is proportional to the number of nodes/states.
5. The efficiency is also improved because the MergedTrie updating operations are based on simple Trie operations.
6. It facilitates the indexation of additional information (e.g. n-grams, phrases, bitexts, biwords or additional data) related to the words stored in all the MergedTries, because it automatically provides word identifiers that different MergedTries can share.

Regarding the practical impact of our research:

1. It has been implemented in C++ and has been comparatively analyzed with the Hash Table, Trie, Crit-bit, ADFA-DAWG, double-array, Double Trie and Minimal-Prefix Trie (with and without string labels), showing an important reduction in memory consumption and number of nodes/edges.
2. Its sequential implementation allows for quite simple and efficient de/serialization of the MergedTrie from/into the disk. It achieves an efficient storage in secondary memory, optimizing the subsequent load times and allowing direct access to the structure when it is stored in secondary memory if it does not fit in the main memory due to the number of keys to be indexed.
3. It requires less space than the original text to be indexed.
4. Regarding temporal efficiency, it especially improves insertion operations, as well as allowing new insertions and updates, in contrast to the static structures that only allow for the search operation.
5. An additional compaction operation is provided in order to improve the computational efficiency of the structure.

In terms of future projects, the authors plan to release the MergedTrie data structure as a public C++ library. Moreover, we will try to improve the temporal efficiency with alternative representations and implementations, especially the issue related to the reduction in the number of collided items. We also plan to extend the MergedTrie structure to the combination of a parameterized n number of Tries, creating a novel structure named the nTrie. Finally, we plan to prove the MergedTrie for representing Suffix Tries in Bioinformatics and Genetic applications, such as DNA sequence processing tasks, which could achieve high overlapping compaction.
