# Correction: MergedTrie: Efficient textual indexing

**DOI:** 10.1371/journal.pone.0217958

**Published:** 2019-05-31

**Authors:** Antonio Ferrández, Jesús Peral

There are errors in the Funding statement. The correct Funding statement is as follows: This study has been partially funded by the ECLIPSE-UA (RTI2018-094283-B-C32) and the RESCATA (TIN2015-65100-R) projects of the Spanish Ministry of Economy and Competitiveness (MINECO) and PROMETEO/2018/089.
